# Reduction in operator radiation exposure during transradial coronary procedures using a simple lead rectangle

**DOI:** 10.1016/j.heliyon.2017.e00254

**Published:** 2017-02-24

**Authors:** Azriel B. Osherov, Sharon Bruoha, Avishag Laish Farkash, Gideon Paul, Ian Orlov, Amos Katz, Jamal Jafari

**Affiliations:** a Division of Cardiology, Barzilai Medical Center, Ashkelon, Israel; b Faculty of Health Sciences, Ben Gurion University of the Negev, Beer-Sheva, Israel; c Beilinson Hospital − Rabin Medical Center, Petach Tikva, Israel

**Keywords:** Medical imaging, Medicine, Health sciences, Cardiology

## Abstract

**Objectives:**

Transradial access for percutaneous coronary intervention (PCI) reduces procedural complications however, there are concerns regarding the potential for increased exposure to ionizing radiation to the primary operator. We evaluated the efficacy of a lead-attenuator in reducing radiation exposure during transradial PCI.

**Methods and results:**

This was a non-randomized, prospective, observational study in which 52 consecutive patients were assigned to either standard operator protection (n = 26) or the addition of the lead attenuator across their abdomen/pelvis (n = 26). In the attenuator group patients were relatively older with a higher prevalence of peripheral vascular disease (67.9 vs 58.7 p = 0.0292 and 12% vs 7.6% p < 0.001 respectively). Despite similar average fluoroscopy times (12.3 ± 9.8 min vs. 9.3 ± 5.4 min, p = 0.175) and average examination doses (111866 ± 80790 vs. 91,268 ± 47916 Gycm^2^, p = 0.2688), the total radiation exposure to the operator, at the thyroid level, was significantly lower when the lead-attenuator was utilized (20.2% p < 0.0001) as compared to the control group. Amongst the 26 patients assigned to the lead-attenuator, there was a significant reduction in measured radiation of 94.5% (p < 0.0001), above as compared to underneath the lead attenuator.

**Conclusions:**

Additional protection with the use of a lead rectangle-attenuator significantly lowered radiation exposure to the primary operator, which may confer long-term benefits in reducing radiation-induced injury.

**Advances in knowledge:**

This is the first paper to show that a simple lead attenuator almost completely reduced the scattered radiation at very close proximity to the patient and should be considered as part of the standard equipment within catheterization laboratories.

## 1 Introduction

The global increase in transradial PCI reflects its proven reduction in procedural bleeding, shorter hospital admissions, improved patient comfort and significant reduction in mortality in patients presenting with ST elevation MI (STEMI) [1, 2, 3, 4, 5]. Recent studies have demonstrated higher radiation exposure associated with radial as compared to femoral access [6, 7]. With the increasing adoption of transradial PCI, the enhanced exposure to scattered radiation has been reported to increase the lifetime risk of both malignancies and cataracts amongst interventional cardiologists [8, 9]. Most of the exposed, scattered radiation comes from the patient’s lower torso i.e from below the umbilicus, which is closest in proximity to the operator [10]. Recent data has demonstrated a significant reduction in scattered radiation with the use of a phantom, covered with a rectangle lead-attenuator [10]. We have designed a prospective, non-randomized study to assess whether a protective lead drape, extending from the region of the umbilicus to mid-thigh, can significantly reduce the dose of radiation exposure to both the operator and colleagues performing transradial coronary angiography and PCI.

## 2 Methods

Study design: Patients referred for non-emergent, radial coronary procedures (angiography and angioplasty) were asked to participate in a non-randomized prospective observational study. All cases were performed by an interventional cardiologist (A.B.O), who has extensive experience in transradial access. The study was approved by the institutional review Helsinki committee and informed consent was obtained from the patients and the primary operator (A.B.O.). Patient population: between January 2014 and July 2014 patients undergoing radial artery procedures at Barzilai Medical Center (Ashkelon, Israel) catheterization laboratory were assigned to two groups: lead rectangle attenuation versus no additional protection with standard operator shielding used for all patients. Inclusion criteria: Age 18–75 years, patients referred for non-emergent coronary procedures (angiography and angioplasty). In addition, hemodynamically stable patients with unstable angina pectoris and non-ST elevation MI (NSTEMI), were also included. Exclusion criteria: ST elevation myocardial infarction (STEMI), suspected left main stem (LMS) coronary artery disease, low ejection fraction <35%, heart failure (NYHA classes III, IV), patients with >10% chance of requiring a change to a femoral access approach (weak radial pulses, low blood pressure <90 mmHg, failed radial access in the past), significant valve disease including severe aortic stenosis and severe mitral regurgitation and pregnancy or women in childbearing age.

### 2.1 Study protocol

**Fig. 1:**
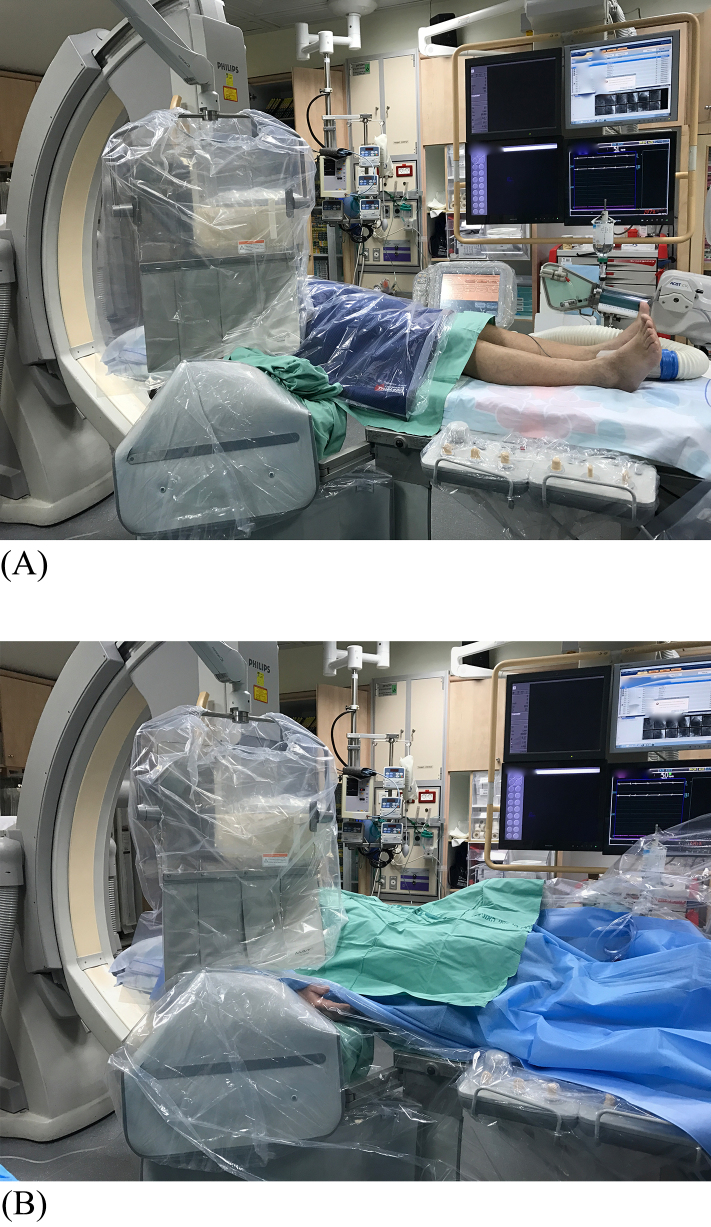
Clinical example of lead rectangle in use for a right radial procedure. (A) The lead attenuator was inserted into a sterile nylon bag. After cleaning both groins, sterile towels were used to cover the groin area, the attenuator then positioned from the umbilicus down. (B) The lead attenuator was covered by a sterile drape. If an urgent femoral puncture is needed, the attenuator can be removed promptly while maintaining a sterile working area.

All procedures were performed by one operator (ABO) and the choice for left versus right sided access was at his discretion although most elderly women and coronary artery bypass graft patients were done through the left radial artery. If radial access failed, the opposite radial or either femoral artery was used at the discretion of the operator. All patient were included in the analysis by intention to treat. Radial access was accomplished by puncture with a 20-gauge needle and insertion of a 5-F or 6-F hydrophilic sheath. Diagnostic and guiding catheters were used at the discretion of the primary operator. Regardless of access site, the operator worked from the right side of the table. In the study group with enhanced protection, a 0.5-mm lead rectangle (60 × 100 cm, 150 kVP) was positioned horizontally from the umbilicus extending inferiorly for 60 cm (Fig. 1A and B). The rectangle was inserted into a sterile nylon bag and positioned after sterilizing the groin area with antiseptic solution. The patient’s hands were placed parallel and on top of the rectangle to prevent it from falling. If needed, minor changes in lead position were taken during caudal views to prevent obscuring the radiographic fields of view. In addition standard personal radiation protective equipment was used: laboratory protection included an overhead-suspended lead acrylic shield (0.5-mm lead equivalent; MAVIG, Munich, Germany) that was lowered to the patient’s abdomen. An under-table pivotal, leaded-side shield (0.5-mm lead equivalent) was mounted to the side of the table. The 17.8-cm upper shield flap was folded up in all cases to provide extra radiation protection.

### 2.2 Cardiac catheterization and PCI

Procedures were performed on a digital single-plane cineangiography unit (Allura Xper; Philips Medical Systems, Best, the Netherlands). Images were acquired with 20-cm magnification and a film speed of 15 frames/s. If required, to optimize image quality, the film speed was increased to 30 frames/s. For each case the fluoroscopy time (FT), air kerma and dose area product (DAP) were recorded. ABO, who had completed more than 3000 radial procedures before the study period, performed all of the cases. Both collimation and frames per second were set at his discretion. Standard dosimeters (Thermo Fisher Scientific Inc. Waltham, MA USA) were used (record level 0.1 mSv). Dosimeters (in triplicates) were placed at the patient’s umbilicus, in both the study and control groups. In the study group an additional set of dosimeters was attached above the lead rectangle itself also at the level of the umbilicus. The third set of dosimeter was attached to the outer side of the operator’s thyroid collar. The dosimeters were sent for analysis every month.

Statistical analysis: Continuous variable are presented as mean ± standard deviation (SD). For comparison between groups, DAP was normalized. The student t-test and analysis of variant were use to compare continuous variables. The X^2^ test was used to compare categorical variables. A 2-sided p < 0.05 was considered statistically significant. Analysis was performed using GraphPad software.

## 3 Results

During the study period exposure to radiation was tested in three areas in 52 patients. Table 1 summarizes the baseline clinical characteristics of the two groups. In the attenuator group patients were relatively older and peripheral vascular disease was more prevalent (67.9 vs 58.7 p = 0.0292 and 12% vs 7.6% p < 0.001 respectively). The groups were well matched regarding body mass index (BMI) and body surface area (BSA). The comparison of operator exposure between the groups is shown in Table 2. There were no significant differences in fluoroscopy time or air kerma. The DAP-normalized operator radiation dose was significantly lower in the attenuator group as compared to control. Dosimeters placed at the thyroid collar showed a 20.2% reduction (95% confidence interval, 0.19 to 0.23, p < 0.0001) with a lead attenuator as compared to standard protection. Within the lead attenuator group itself, there was a significant reduction (94.5%, p < 0.0001) in measured radiation below as compared to above the lead rectangle (Table 3).

## 4 Discussion

In this study we have demonstrated that a non-disposable lead attenuator significantly reduced the dose of radiation to the primary operator. A simple, cost-effective lead rectangle placed across the patient’s lower abdomen and upper thighs reduced measured radiation by >94% (Table 3). This is the first clinical study to show a dramatic reduction of radiation in concordance with the results of the mentioned preclinical study [10]. During fluoroscopic imaging, most x-ray photons traverse the patient to reach the detector to produce the diagnostic image whilst less then 1% of X-ray photons are absorbed by, or scattered from the patient. As a result the distribution of scattered radiation around the patient that results in personnel exposure is highly dependent on the position of the x-ray source, which is varied throughout the procedure to obtain different views of the anatomy of interest [9]. Despite standard safety precautions such as the use of a lead shield between the patient and operator, some operators are exposed to higher doses of radiation (greater than the permitted 20 mSv per year), which increases the risk of both stochastic and deterministic side effects [9]. In our opinion, the lower doses recorded by the thyroid dosimeters likely reflects the fact that they measure scattered radiation from the patient’s upper torso which is not covered by the lead rectangle. Furthermore using standard protection with an overhead-suspended lead acrylic shield, (which has a 99% efficacy, according to manufacturer information or at least 80% efficacy, according to clinical measurements [11]) the dosimeters placed at the thyroid collar would usually detect relatively low readings, which are close in fact to their actual sensitivity (0.1 mSv). In this regard even using cumulative readings from several consecutive patients could still be prone to a statistical error.

In addition, we have shown that the lead attenuator did not interfere with the field of radiation required for fluoroscopy (the upper border sat at the patient’s umbilicus and shown to be the optimal position for caudal views). Of note, if radial access was unsuccessful, it was still possible to proceed directly to a transfemoral approach, as the lead rectangle was dressed within a standard, sterile nylon sheath.

At the time of the study, no previously published studies were available with sample size calculations. A small heterogeneous group of 52 all-comers were recruited and whilst this represented a relatively small sample size, it was sufficient to show significant differences in radiation exposure. Further studies may be best focused on a single side approach and for a specific PCI procedure e.g. single vessel angioplasty and radiation views. In such a small consecutive patients cohort, although basic and procedural characteristics were similar (Table 1) it is difficult to obtain two groups that are well matched for procedural complexity.

Few studies have formally assessed whether patients treated by the radial approach are exposed to additional radiation and as a consequence the performing operators, compared with PCI performed by the femoral approach [6, 12, 13]. The main reason underlying this is the possible longer fluoroscopy time associated with the transradial approach and operator being positioned closer to the patient’s torso [12, 13, 14].

Jolly et al. [13], showed that radiation dose, as measured by air kerma, was nominally higher with radial versus femoral access, but differences were present only in lower-volume centers with corresponding lower-volume operators. Similarly the lowest radiation dose and air kerma were recorded, irrespective of which access site was used, at high-volume radial centers. However, the assumption that the radiation dose to the operator is mainly influenced by the relative experience of an interventionist cardiologist and not by the access site remains controversial [6]. Politi et al. [15] have demonstrated that a disposable bismuth-barium radiation shield drape ‘Radpad’ decreased operator dose by 23%. In their study they used a 35 × 40 cm drape shield that was placed on the patient’s arm, adjacent to the site of sheath insertion and extended medially to the patient’s body. The significant reduction in radiation exposure seen in our study is attributed to the size of the lead drape whose area is 4.2 times larger than the Radpad. The similar results detected at the thyroid level might be due to different views used in both studies, which can have a significant impact regarding the amount of radiation detected further encouraging our interest in the use of the lead attenuator.

Recently Iqtidar et al. [16] showed that measured exposure was reduced with enhanced pelvic shielding at all dosimeter sites except at the secondary operator's collar (both at the left and right sides) and at the primary operator's collar from the right side. In contrast to their study, we used triplicates of dosimeters at each tested site for a group of consecutive patients, which reduces the probability of potential error. In addition, an experienced operator performed all the procedures alone, eliminating operator-related factors again reducing the chance for a potential error.

Lange et al. [17] demonstrated that the pelvic lead-shield is a highly effective protection device reducing radiation dose by 58.5% in patients undergoing transradial coronary angiography. They recorded the operator radiation dose at the beginning and the end of each procedure using an electronic Geiger Muller radiation device. A great effort was made to calibrate the dosimeters to account for low-energy scatter radiation. The average radiation detected per procedure was as low as 9.0+/−5.4 μSv. In our experience, when dosimeter readings are very low, there is a higher chance for a technical error hence the need for duplicates or triplicates that can add additional data resulting in a lower margin of error. In addition, the pelvic lead shield was designed to reduce scattered radiation while enabling both radial and femoral access. As a result, in order to allow femoral access, the pelvic lead shield leaves both groins exposed increasing risk of potential infection, hence the requirement of a sterile nylon cover specifically designed for the pelvic shield. In our study, a simple sterile nylon bag (similar in size to the one covering the ceiling attenuator) was used to cover the lead rectangle and is being replaced between procedures. This makes a routine use of the lead rectangle more cost effective.

An experienced team led by Roguin et al. [18], recently showed a statistically significant reduction by 68% at the thyroid collar level, in 332 randomly assigned patients. However their study showed a two-fold increase in back scatter using the shield. Based on these results, using a simple calculation dividing the total backscatter radiation by the number of patients (13.5 μS per patient), we would not anticipate any clinical significance related to this backscatter. In contrast to Roguin et al., phantom studies performed by Osherov et al. [10] were unable to demonstrate any significant increase in back scattered radiation. The concern over increased radiation exposure identified from these studies, however, was non significant. In addition it must be emphasized that scattered radiation is known to be only a small fraction of the total radiation that patients are exposed to (less then 0.5%). In conclusion back scattered radiation does not appear to be of significant clinical significance.

Assessing data from preclinical radiation studies [10], the main obstacle to the correct measurement and assessment of the magnitude of backscattered radiation is the accurate positioning of dosimeters underneath the lead rectangle (small differences in dosimeter position can have a significant influence on readings). In our opinion, accurate and reproducible results can be only attained using phantom radiation studies. According to the above we suggest that more phantom studies are needed to accurately address this important issue of backscatter radiation.

In a recently published clinical study, Savage et al. [19] evaluated the use of a new shielding device, the Zgrav (Zero Gravity system), a suspended personal radiation protection system, in comparison to conventional operator protection (lead-apron and shields) during interventional fluoroscopy. The suspended system provided operator protection during various interventional fluoroscopy procedures, reducing radiation exposure by 87–100% to the eyes, head, neck, humerus and tibia. The Zgrav has several limitations however: it only protects the primary operator and does not reduce backscatter to other personnel in the room; there is no protection for the arms and hands of the operator, exposing the upper limbs to high levels of radiation; the Zgrav device is expensive (five hundred times more expensive than a 60 × 100 lead rectangle) and limits free movement of the primary operator. All these factors make the Zgrav less appealing for use in the catheterization laboratory.

Our data showed a significant fall in DAP from 1.059 to 0.845, however the average fluoroscopy time per patient was increased from 9.5 min to 12.53 min (Table 2). Although the increase in fluoroscopy time was non-significant, we are concerned that the interventional cardiologist might use higher doses of radiation and longer fluoroscopy times because he or she may feel “more protected”. The net effect of improving operator’s safety on patient exposure is complex. It is unclear if the increase in fluoroscopy time was due to procedural requirements or whether the interventional cardiologist felt safer with the lead rectangle. To overcome the above we suggest increasing the use of beam collimation [9], which will decrease the radiation delivered to the patients and medical personnel in the room. Collimating to the area of interest reduces exposure by reducing the volume of tissue that is irradiated. As a result, it also reduces scattered radiation within the patient and in the procedure room, reducing both patient and personnel exposure. This important issue should be addressed with further clinical studies.

### 4.1 Study limitation

The patients in our study were not randomized to standard protection only or the addition of the lead rectangle. Although this could influence our results, by normalizing for DAP any significant differences are likely to have been corrected. For the assessment of the relative efficacy of an added radiation protection device, the DAP-normalized operator dose, has been advocated and was applied in our study. Although DAP is commonly used in studies, it is influenced by several factors, mainly dose and area of radiation which are highly dependent on patient characteristics and procedural requirements hence more studies are needed to verify our results. Our study was not designed to analyze uniform and highly comparable cohorts, and no adjustment was made for differences in procedural factors. Due to variability between different operators regarding fluoroscopy times, angles of the C arm, etc. which influences significantly DAP and dosimeter readings, the study was done by a single experienced operator. Hence, the relative efficacy of radiation protection provided by a pelvic lead attenuator may vary in different selected cohorts with different operators and coronary interventional procedures.

Our study was not designed to assess the magnitude of backscatter, which can be tested in phantom or larger clinical studies. As backscatter is generated from less then 0.5% of the total radiation delivered, it is unlikely to be of any clinical significance. Also details of the fluoroscopy angles used during each case were not assessed and this could have a significant effect on operator dose and would vary between patients.

Covering the upper torso with a lead attenuator might confer additional scattered radiation protection especially with cranial right coronary interventions or distal left posterior descending artery. A study for clarifying this important issue is planned.

Conclusions: The use of a simple, novel lead-attenuator significantly reduces radiation exposure to the primary operator performing transradial PCI, which may confer long-term benefits in reducing radiation-induced injury.

## Declarations

### Author contribution statement

Azriel B. Osherov: Conceived and designed the experiments; Performed the experiments; Analyzed and interpreted the data; Contributed reagents, materials, analysis tools or data; Wrote the paper.

Sharon Bruoha: Analyzed and interpreted the data; Contributed reagents, materials, analysis tools or data.

Avishag Laish Farkash: Contributed reagents, materials, analysis tools or data; Wrote the paper.

Gideon Paul: Analyzed and interpreted the data; Contributed reagents, materials, analysis tools or data; Wrote the paper.

Ian Orlov, Amos Katz: Contributed reagents, materials, analysis tools or data.

Jamal Jafari: Contributed reagents, materials, analysis tools or data; Wrote the paper.

### Funding statement

This research did not receive any specific grant from funding agencies in the public, commercial, or not-for-profit sectors.

### Competing interest statement

The authors declare the following conflict of interests: Azriel B. Osherov has applied for a patent based on this aforementioned lead attenuator.

### Additional information

No additional information is available for this paper.

## Figures and Tables

**Table 1:** Patients and procedure characteristics.

	Enhanced Protection (n = 26)	Standard Protection (n = 26)	P value
Age (y)	67.9	58.7	0.0292
Male (%)	72	84.6	0.2152
BMI (kg/m^2^)	28.8	29.7	0.5010
BSA (m^2^)	1.927	2.013	0.1647
Hypertension (%)	76	69	0.5794
Hyperlipidemia (%)	80	80.7	0.9494
Diabetes (%)	48	50	0.8853
Tobacco use (%)	52	38	0.3103
PVD (%)	12	7.6	<0.0001
Prior PCI (%)	40	30.7	0.453
Prior Bypass surgery (%)	4	0	0.3029
PCI (%)	64	61.5	0.8521
Left radial (%)	30.7	46.1	0.25428
Right radial (%)	69.3	53.9	0.25428

Abbreviations: BMI, Body mass index; PVD, peripheral vascular disease. P constitutes P value for enhanced versus standard shielding.

**Table 2:** Comparison of operator exposure between attenuator and control group.

	Enhanced Protection (n = 26)	Standard protection (n = 26)	P value
Total Fluoroscopy time, hours	5:26 +\-0:05	4:07 +\-0:09	0.273
Average Fluoroscopy time per patient	12.53 min	9.5 min	
DAP, Gy*cm^2^	111866 +\-80790	91268 +\-47916	0.231
Operator radiation dose, mSv, mean	2.864	1.304	0.187
Air kerma, mean, mGy	1666.28	1344	0.183
DAP-normalized operator radiation dose	0.845	1.059	<0.0001 CI 95%

Abbreviations: DAP, dose area product.

**Table 3:** Exposure detected underneath and above the attenuator compare to control at umbilicus area.

Dosimeter site	Enhanced Protection (n = 26)	Standard Protection (n = 26)	P value
Umbilicus (below attenuator)*	28.78	13.4	0.0014
Average per patient	1.1	0.51	
Umbilicus (above attenuator)*	1.84	-	
Average per patient	0.07		

Reported results are in miliSievert.

* Total dose for all cohort.
